# The perception of genetic diseases and premarital screening tests in the central region of Saudi Arabia

**DOI:** 10.1186/s12889-024-19029-0

**Published:** 2024-06-10

**Authors:** Mariam M. Al Eissa, Fahad Almsned, Reem R. Alkharji, Yousif M. Aldossary, Raghad AlQurashi, Esraa A. Hawsa, Sahar M. AlDosari, Amerh S. Alqahtani, Raniah S. Alotibi, Raed Farzan, Reema Alduaiji, Suha M. Sulimani, Shaker A. Alomary, Abdullah M. Assiri

**Affiliations:** 1 Molecular Genetics Department, Molecular Genetics Laboratory, Public Health Laboratory, Public Health Authority, Riyadh, Saudi Arabia; 2 Medical School, AlFaisal University, Riyadh, Saudi Arabia; 3 The Computational Sciences Department at the Centre for Genomic Medicine (CGM), King Faisal Specialist Hospital & Research Center, Riyadh, Saudi Arabia; 4 Research Center, King Fahad Specialist Hospital in Dammam (KFSH-D), Dammam, Saudi Arabia; 5 Population Health Management, Eastern Health Cluster, Dammam, 32253 Saudi Arabia; 6 School of Systems Biology, George Mason University, Fairfax, Virginia USA; 7 Research Department, Health Sciences Research Centre, Princess Nourah Bint Abdulrahman University, Riyadh, Saudi Arabia; 8 Medical Laboratory Sciences Department, Assistant Professor and Consultant of Molecular Genetics, Majmaah University, Riyadh, Saudi Arabia; 9 Department of Medical Genetics, CHU de Lyon, Lyon, France; 10 Department of Clinical Laboratory Sciences, College of Applied Medical Sciences, King Saud Bin Abdulaziz University for Health Sciences (KSAU-HS), Riyadh, Saudi Arabia; 11 King Abdullah International Medical Research Center (KAIMRC), Riyadh, Saudi Arabia; 12 Department of Clinical Laboratory Sciences, College of Applied Medical Sciences, King Saud University, Riyadh, 11433 Saudi Arabia; 13 Center of Excellence in Biotechnology Research, College of Applied Medical Sciences, King Saud University, Riyadh, 11433 Saudi Arabia; 14 Chair of Medical and Molecular Genetics Research, King Saud University, Riyadh, 11433 Saudi Arabia; 15 John Hopkins Aramco Healthcare, Dhahran, Saudi Arabia; 16 Healthy Marriage Program, Deputyship of Public Health, Ministry of Health, Riyadh, Saudi Arabia; 17 Directorate of Health Programs and Chronic Diseases, Ministry of Health, Riyadh, 11176 Saudi Arabia; 18 Assistant Deputyship of Preventive Health, Ministry of Health, Riyadh, Saudi Arabia

**Keywords:** Premarital screening, Saudi Arabia, Genetics screening, Genetic diseases

## Abstract

The prevalence of consanguineous marriages (CMs) varies worldwide from one country to another. However, the Middle East stands out as a region with a notably high rate of CMs. CM is particularly widespread in Saudi Arabia, where the prevalence of autosomal recessive genetic diseases has increased. This study aims to identify the Saudi population’s awareness of genetic diseases and premarital screening tests (PMSTs). It also seeks to understand couples’ perceptions of genetic diseases before and after marriage and their attitudes towards PMSTs and genetic counselling (GC) in reducing the risk of CM. Through the administration of online questionnaires, this cross-sectional study surveyed 2,057 participants to assess their awareness of genetic diseases and their understanding of testing and preventive measures for inherited diseases. Descriptive analysis, nonparametric chi-square tests and logistic regressions were performed to assess the association of categorical responses. This study included 2,035 Saudi Arabian respondents. A significant correlation was found between positive family history and partner selection (*p* = 0.001), as well as between partnering within the same tribe (*p* = 0.000139), with a different tribe (*p* = 0.000138) and from another family (*p* = 0.000489). About 91.3% of participants expressed agreement regarding the need to enhance public awareness and knowledge concerning genetic disorders, while 87% agreed that increased government regulations are required to prevent the spread of genetic diseases in affected families. Despite increased awareness of genetic diseases and PMSTs, there appears to be a lack of understanding regarding the limitations of PMSTs. The persistently high rate of CM underscores the challenge of altering marriage customs. Further governmental efforts are required to promote awareness of alternative reproductive options, establish new regulations and expand screening programmes.

## Introduction

The estimated prevalence of congenital conditions, including single-gene diseases and childhood-related genetic factors, is 37/1,000 [1]. Globally, the prevalence of single-gene disease is 9/1,000 live births, with the Middle East exhibiting the highest prevalence at 20/1,000 births compared to Africa and South East Asia, where it hovers around 9/1,000 births [2]. Under-five years old mortality due to genetic disorders is reported at 15/1,000 live births [2]. Mortality rates in the neonatal and postnatal periods stemming from genetic-related conditions are notably elevated, compared to other causes of mortality, at 5% and 8%, respectively [3]. Consanguinity directly influences the prevalence of genetic disorders, with variations observed between unrelated consanguineous autosomal recessive disorders and consanguinity-related autosomal recessive disorders, ranging from 1.84/1,000 total births to 6.5 times the coefficient of consanguinity multiplied by 100/1,000 total births, respectively [4]. High consanguinity rates in Saudi Arabia (SA) increase the prevalence of the Mendelian autosomal recessive phenotype in 77% of Saudis [5, 6]. This leads to a high carrier frequency of diseases, such as cystic fibrosis and sickle cell anaemia, in the Saudi population [7, 8] and an increase in the rate of autosomal recessive genetic disorders [9–11]. The prevalence of consanguineous marriages (CMs) varies globally from one country to another. The Middle East exhibits the highest prevalence rate of CM, encompassing approximately one-fifth of the world’s population [12]. According to the Saudi Arabian General Authority for Statistics, the Ministry of Justice issued marriage certificates to 137,918 couples in 2019 [13]. Estimates of CM prevalence vary regionally within Saudi Arabia. Approximately half of the Saudi population is involved in CM, with half of these CMs involving first cousins [14].

Given the high disease burden of genetic disorders due to CM, the decision regarding pre-marriage partners in Nigeria did not significantly change despite awareness of the future risk of having affected offspring with autosomal recessive disorders, with one-third of individuals willing to proceed with the marriage despite this knowledge, similar attitudes were observed in Saudi Arabia [15].

Tribalism has long been a fundamental aspect of the social, cultural and economic fabric in the Arabian Peninsula. Saudi tribes have historically prioritised the preservation of community cohesion, identity and patrimony by encouraging marriage alliances within the tribe, lineage or sub-tribe [14]. Consequently, the high prevalence of CM has been culturally justified and accepted among the young educated Saudi generation [16], leading to an elevated incidence of homozygous autosomal recessive disorders in Saudi Arabia and the preservation of several founder effect variants [17, 18].

Due to the high prevalence of hereditary disorders, the Saudi premarital screening programme was established in 2004 as a mandatory screening initiative for couples planning to marry [19]. The screening protocol targets diseases that pose a significant health burden on both the government and individuals, aiding in the assessment of future family planning decisions. Priority diseases include haemoglobinopathies, such as thalassaemia and sickle cell anaemia (SCA), and infectious diseases, such as hepatitis B and C and HIV. The objective of this screening test is to provide carrier or affected couples with the option to proceed with the marriage while fully understanding the likelihood of their children being affected by the inherited condition and to refer them to a genetic counsellor (GC) for further guidance [20].

The Saudi National Transformation Programme, launched in 2016 by the Saudi Arabian government, aims to ensure that the Kingdom’s Vision 2030 is fulfilled; an essential health aspect of this programme is to ensure prevention and early intervention [21]. The screening programme introduces several advantages to the community, including preventing future complications, assessing early detection and fulfilling risk assessments [18, 22].

The government of Saudi Arabia has made a considerable effort to reduce the risk of marriages between high-risk couples. However, despite this information, 90% of the couples proceed with marriage despite the potential risk of having affected offspring [19]. The community seems to be aware of the consequences of high-risk marriages between at-risk individuals, particularly the high probability of having an affected child. This observation was made in a study conducted between 2004 and 2009, in which the overall prevalence of β-thalassaemia was reduced following a substantial decrease in high-risk marriages [23]. This reduction, compared to the findings from a previous study by AlHamdan et al., suggests that the premarital screening programme has effectively raised awareness among the population [24].

Since the official launch of the Saudi premarital screening programme, few studies have examined its efficiency in reducing the rate of haemoglobinopathies, altering the attitudes of high-risk couples towards marriage [24] and assessing the awareness of the premarital screening test and attitudes towards undergoing the test [16]. To date, there has been no assessment of Saudi citizens’ awareness and knowledge regarding inherited disorders other than thalassaemia and SCA, their understanding and implementation of preventive measures, their criteria for selecting partners and how hereditary disorders, not limited to haemoglobinopathies, might influence their decisions. Furthermore, the factors driving Saudis to seek genetic counselling services and undergo genetic investigations have not been previously addressed. We expect that the outcome of this study will inform decision-makers about improving the Saudi premarital screening programme and ensuring the effectiveness of health education among Saudi citizens.

The significance of understanding the relationship between genetic profiles and pre-marriage partner decisions in Saudi Arabia stems from the high prevalence of genetic disorders in the Saudi population. This study holds critical importance due to the unique social, cultural and religious contexts in Saudi Arabia, where consanguineous marriages are common and significantly influence genetic disorder rates among offspring [16–18]. The policy relevance of the study exploring the relationship between genetic profiles and pre-marriage partner decisions, especially in the context of Saudi Arabia, is multifaceted and significant. This study provides valuable insights that can inform the development, implementation and refinement of health, social and educational policies aimed at improving public health outcomes, enhancing genetic literacy and supporting informed reproductive choices. Here are key areas of policy relevance: Findings underscore the importance of premarital screening (PMS) programmes as effective tools for reducing the prevalence of genetic disorders. Policies can be developed to expand the scope of PMS programmes to include a wider range of genetic conditions, ensuring that couples are well-informed about their potential genetic risks before marriage by integrating GC. Policies could support the development of educational materials and programmes in variable settings to reach a broad audience and enhance public knowledge about the risk of hereditary disorders associated with CM practices and the benefits of participating in PMS programmes.

Given the cultural and religious sensitivities surrounding reproductive decisions and genetic testing in Saudi Arabia [18], policies must be designed to respect these values while promoting public health. This could involve engaging with religious and community leaders to endorse and advocate for genetic screening and counselling services. For families with genetic disorders, policies could provide support mechanisms, including financial assistance, healthcare services and social support programs, to help manage these conditions effectively and improve their quality of life. Encouraging further research on genetic diseases prevalent in Saudi Arabia and the impact of genetic counselling and PMS programmes can help in continuously updating and refining policy decisions based on the latest scientific evidence. The development of comprehensive legal and ethical frameworks to govern genetic testing and counselling practices is crucial. Integrating both theoretical and conceptual frameworks into policy analysis regarding the reduction of genetic disorders through informed reproductive choices in Saudi Arabia adds depth and clarity to the study. The theoretical framework introduces theories that explain why certain policies might be effective in reducing genetic disorders and improving public health. For instance, behavioural change theories might elucidate why individuals decide for or against genetic counselling and screening. Health belief models could explain how perceptions of susceptibility and severity of genetic disorders influence people’s readiness to engage in premarital screening. A conceptual framework for this policy analysis would identify key variables, such as genetic disorder prevalence rates, consanguineous marriage rates, public awareness levels and the accessibility of genetic counselling and PMS programs. It outlines the expected relationships among these variables, such as how increased accessibility to genetic counselling might influence the rates of CMs and, subsequently, affect the prevalence of genetic disorders [25, 26].

This study assesses the community’s awareness of genetic diseases and appraises the perception of avoiding or reducing inherited diseases. It evaluates the awareness of premarital screening tests (PMSTs) and other genetic tests among families affected by inherited genetic diseases. Additionally, the research investigates couples’ preferences in selecting partners, their perceptions before or after marriage regarding options for preventing inherited diseases, and their willingness to seek genetic counselling before engagement. The study also explores public opinions on the factors influencing these decisions and the motivation behind pursuing genetic testing.

## Materials and methods

### Ethical approval

This study complied with the Declaration of Helsinki and was approved by the Institutional Review Board at the Ministry of Health ethics committee, Saudi Arabia, with approval number IRB-22-13E and national registry number NCBE-KACST, KSA(H-01-r-009).

### Study design

The study was a cross-sectional (online) survey via a Google form entitled ‘Genetic profile effect on pre-marriage partner choice in Saudi Arabia’. The survey was designed based on extant literature; a few items were adapted from previously validated and published questionnaires [16]. Other items were designed based on experts’ comments, previous recommendations and study questions. It was designed first in English and translated into Arabic by bilingual translators and reviewed by the research team and external participants to suit the general population. External experts evaluated the survey to check the validity of its content and language. The reliability of the survey was tested using Cronbach’s alpha, which was 0.73 for all items. Participants were informed that the survey would take approximately 10–15 min to complete. Simple random sampling served as the primary strategy for participant selection in our study. The survey was distributed in an online Arabic version through two platforms: premarital preventive clinics under the Ministry of Health and social media channels such as WhatsApp. To prevent duplicate or multiple submissions, only one submission per IP address was permitted.

### Study variables

The survey consisted of four parts. The first part covered demographic variables, including gender, age, educational level, marital status, number of marriages, number of children, region, employment and family income. The second part focused on assessing participants’ knowledge about genetic diseases, such as personal and/or family history, awareness of hereditary disorders or genetic counselling clinics and previous attendance at genetic counselling sessions. Additionally, it gauged participants’ awareness of the Saudi premarital screening programme, including their understanding of its benefits and its limited coverage to only two haematological disorders. The third part of the survey solicited information on affected family members, if applicable, including offspring, partners or remote relatives. It also enquired about the kinship between partners, specifying whether they were first-degree or second-degree relatives whether the marriage occurred within the same tribe, outside the tribe, or involved a non-Arab Saudi. The timeframe of marriages spanned from the 1970s to 2020. Furthermore, it explored participants’ choice of partner, distinguishing between personal choice and arranged marriage. For single individuals, it investigated their preference for choosing a future partner, whether by personal choice or through an arranged marriage.

The survey included questions about the possibility of having offspring with inherited disorders. Participants were asked whether they would proceed with the marriage, knowing that it might yield an affected child, or cancel the wedding if a partner harbours an autosomal dominant disorder. Carrier couples with autosomal recessive disorders were asked whether they would find a new partner, consult a genetic counsellor or proceed with the marriage. Participants were asked whether they had heard of preimplantation genetic diagnosis (PGD), whether one partner was a confirmed carrier for an inherited disorder or whether the other partner was willing to be tested to ensure the absence of the same condition before marriage. Couples with histories of genetic disorders were asked whether they were willing to visit the genetic counsellor’s clinic and follow the counsellor’s recommendations. The couples were also asked whether they would be willing, in the future, to proceed with the medical termination of pregnancy whenever it was necessary.

The fourth part of the survey included public opinion regarding the factors that affected the participants’ decisions to proceed with genetic testing. Variables encompassed whether the Saudi premarital screening was deemed sufficient, financial constraints hindering access to genetic testing, reluctance due to social stigma and societal awareness of an individual being a carrier of a genetic disease that could affect their future children. Other variables included unawareness of being a genetic disease carrier, lack of knowledge about a family history of genetic disease, uncertainty regarding whether genetic testing can have an impact on the offspring, lack of awareness about genetic testing and unfamiliarity with the role of genetic counselling clinics.

Additional factors influencing motivations for undergoing genetic testing, either individually or as a couple, included awareness campaigns to improve knowledge about genetic diseases, comprehending alternative options to prevent the transmission of inherited diseases to future generations, a desire to safeguard one’s offspring, sharing experiences with others, affordability of genetic testing, accessibility of genetic testing coverage through health insurance, alleviating social constraints associated with arranged marriages within a clan or tribe and governmental regulations to prevent the spread of genetic diseases within affected families.

### Inclusion and exclusion criteria

This study was conducted among Saudi Arabians aged over 18 years, including both married and unmarried individuals who are literate. The exclusion criteria included prisoners, individuals with physical or mental disabilities and those belonging to low-income households.

### Sample size and data collection

The survey was conducted from March to December 2022, ensuring that all collected data were anonymised and securely stored within the Public Health Authority’s databases, accessible solely by the research team. Given the scant information available on the target population, we leveraged the Slovin formula (*n* = N/(1 + Ne^2^)) to ascertain the minimal sample size required for our study. With an initial population size estimated at approximately 3,5000,000 and a margin of error set at 0.5, we calculated a necessary sample size of 400 [27]. The survey was designed to extend the data collection period if needed to maintain a sample size above 400. A total of 2,700 participants ultimately participated in the survey, substantially exceeding the minimal sample size requirement and enhancing the robustness of our study findings.

### Statistical analyses

A total of 2,700 participants were recruited for this study, and the response rate was 76%. Of these, 2,057 individuals completed the online survey. Incomplete responses (*n* = 22) were excluded from the study, leaving 2,035 participants comprising 1,279 females (62.85%) and 756 males (37.15%). The analyses were mainly descriptive. Where appropriate, nonparametric chi-square tests and logistic regressions were performed to assess the association of the categorical responses. IBM Statistical Package for the Social Sciences (SPSS) version 29.0 was used to check the validity of the survey using Cronbach’s alpha. The rest of the statistical analyses were conducted using the R Statistical Programming Language (v 4.2.2) [28]. This included the application of chi-square tests, logistic regression models and Cramér’s V tests, implemented using the relevant R package. Plots were generated using the ggplot2 (v. 3.3.3) (https://cran.r-project.org/web/packages/ggplot2/index.html) and the plotrix (v. 3.8–1) (https://cran.r-project.org/web/packages/plotrix/index.html) R packages [29, 30]. In addition, we used the dplyr (v. 1.0.2) (https://cran.r-project.org/web/packages/dplyr/index.html) and the tidyverse (v. 1.3.0) (https://cran.r-project.org/web/packages/dplyr/index.html) R packages for data manipulation [31, 32]. The Perception section includes questions on personal beliefs and societal implications of genetic diseases. It simplifies by retaining only affirmative ‘Yes’ responses. The questions are converted into binary variables (‘0’ or ‘1’), with ‘1’ indicating a positive perception. These are totaled to categorize perception scores as ‘poor’ (0–6) or ‘good’ (7–10). The analysis will employ bivariate models to identify initial associations and multivariate models adjusted for significant variables at the 10% level to determine the adjusted impacts. This methodology robustly examines the influence of perceptions of genetic diseases on premarital screening decisions in the Central Region of Saudi Arabia.

## Results

### Sample characteristics

**Fig. 1:**
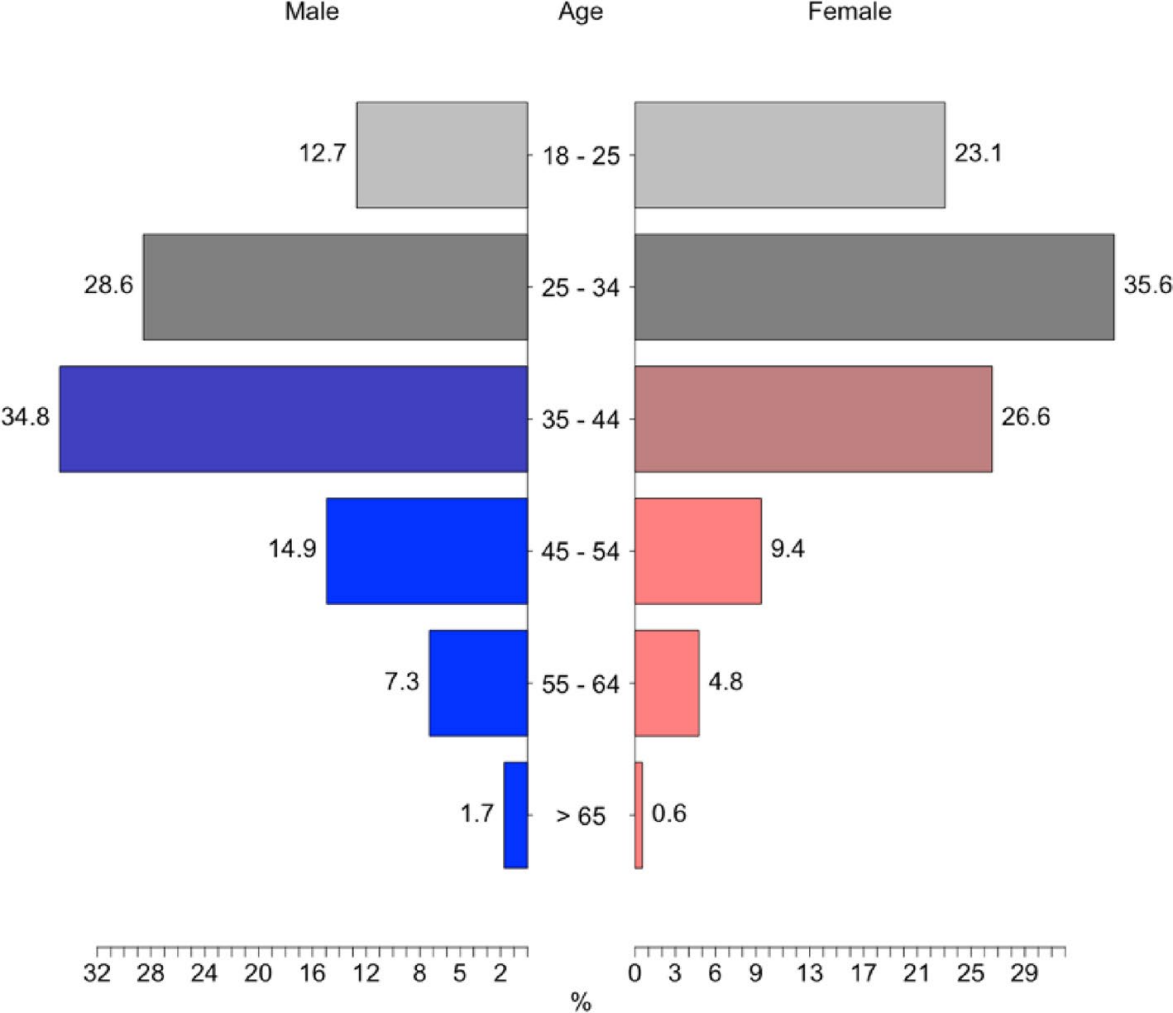
Distribution of participants’ ages comparing males and females

This study included 2,035 completed responses, with 46% of participants from the central region of Saudi Arabia. The mean age of the sample was 29.7 years (Fig. 1). Approximately 63% of the study participants were female, and 37.05% were male. Moreover, 88.27% of the participants were university students/university graduates. About 1.03% of the participants attended lower than high school. About 55.5% of the respondents were married. Table 1 summarises respondents’ demographic information and background characteristics, including age, employment, education levels, marital status and geographical region.

**Table 1:** Demographic data of participants

Category	Variable	N	%
Gender	Male	756	37.15
	Female	1279	62.85
Age group	18–24	391	19.21
	25–34	672	33.02
	35–44	603	29.63
	45–54	233	11.45
	55–65	116	5.70
	> 65	20	0.98
Employment	Employee	1258	61.82
	Not working	777	38.18
Education	Illiterate	1	0.05
	Elementary	4	0.2
	Intermediate	1	0.64
	Secondary	214	10.52
	Bachelor	1166	57.15
	Graduate	640	31.45
Province	Central	935	45.95
	Eastern	276	13.56
	Northern	167	8.21
	Southern	179	8.80
	Western	478	23.49
Income	< 5,000 SAR	575	28.26
	5,000–9,999	390	19.16
	10,000–14,999	465	22.85
	> 15,000 SAR	605	29.73
Marital status	Divorced	112	5.50
	Married	1133	55.68
	No response	1	0.05
	Other	1	0.05
	Single	776	38.13
	Widow	12	0.59
Number of marriages	First	1178	57.89
	Second	119	5.85
	Third	18	0.88
	Fourth	7	0.34
	Single	713	35.04
Number of children	< 2	1063	52.24
	2–5	816	40.10
	6–9	148	7.27
	> 9	8	0.39

### Awareness of genetic diseases

Responses to determine the level of awareness of genetic diseases revealed that most respondents (*n* = 2,046; 97.6%) were aware of their genetic disorders, while 61% were unaware of genetic testing. In assessing the determinants of knowledge regarding genetic disorders, our multivariable logistic regression analysis revealed several notable associations. Graduates were significantly more likely to know about genetic disorders, as indicated by the positive coefficient (Estimate: 0.7961, Std. Error: 0.2518, z-value: 3.161, *p*-value: 0.00157). Conversely, participants with elementary-level education were less likely to possess such knowledge, as suggested by a negative coefficient (Estimate: − 3.25382, Std. Error: 1.18209, z-value: − 2.753, *p*-value: 0.00591) (Supplementary Table 7).

### Awareness of PMSTs and genetic counselling

Responses to identifying the level of awareness between the differences in premarital and genetic screening tests revealed that 68% believed that PMSTs were sufficient to save their families from genetic diseases. About 27% of respondents believed that PMSTs covered all genetic diseases, while 61% knew that premarital screening included only two haematological genetic diseases. Most of the participants recognised the benefits of PMSTs and were willing to undergo screening, even if they were carriers or had a history of genetic diseases, as indicated in Table 2.

**Table 2:** Level of awareness of genetic diseases

Category	Variable	N	%	Chi-square test
Heard about genetic diseases	Yes	1987	97.64	χ^2^ = 1847.5, p < 0.001
	No	49	2.36	
Heard about genetic counseling	Yes	1154	56.71	χ^2^ = 36.62, p < 0.001
	No	881	43.29	
Ever visited a genetic counseling clinic	Yes	150	13.00	χ^2^ = 1478.4, p < 0.001
	No response	9	0.78	
	No	995	86.22	
Ever tested for genetic diseases	Clinical diagnosis with a genetic disorder	129	6.34	χ^2^ = 1673.3, p < 0.001
	No	1119	54.99	
	Other	1	0.05	
	Premarital testing	786	38.62	
Benefit of premarital screening tests’ recognition	Yes	1947	95.68	χ^2^ = 1698.2, p < 0.001
	No	88	4.32	
Any family history of genetic diseases	Yes	805	39.56	χ^2^ = 88.76, p < 0.001
	No	1230	60.44	
Preferred marriage arrangement	Already married (arranged marriage)	1047	51.45	χ^2^ = 507.6, p < 0.001
	Prefer to choose/pre-marriage relation	759	37.30	
	Not married (prefer arranged marriage)	229	11.25	
Relative status of partner	Single	652	32.04	χ^2^ = 761.87, p < 0.001
	2nd degree (first cousin)	286	14.05	
	3rd degree (second or third cousin)	179	8.80	
	Different family	286	14.05	
	Different tribe	361	17.74	
	From the tribe	248	12.19	
	Saudi non-Arab	23	1.13	
Last marriage	70 s and 80 s	89	4.37	χ^2^ = 849.26, p < 0.001
	90 s and post-2000s	1139	55.97	
	Soon	807	39.66	
Premarital screening covers genetic diseases	Yes	1488	73.12	χ^2^ = 435.13, p < 0.001
	No	547	26.88	
Premarital screening only includes two hematological genetic diseases	Yes	1245	61.18	χ^2^ = 101.73, p < 0.001
	No	790	38.82	
Partner at risk of having a baby with a high likelihood of genetic disease	Yes	907	44.57	χ^2^ = 24, p < 0.001
	No	1128	55.43	
Partner’s willingness to test for genetic diseases before marriage, if aware of family history	Yes	1956	96.12	χ^2^ = 1731.3, p < 0.001
	No	79	3.88	
Wedding cancellation during courtship because of awareness of a genetic disorder that is transferrable to the offspring	Yes	1811	89.99	χ^2^ = 1237.6 p < 0.001
	No	224	11.01	
Adherence to the counselor's recommendation if either partner has a history of genetic diseases	Yes	1920	94.35	χ^2^ = 1601, p < 0.001
	No	115	5.65	
Proceed with marriage despite having the risk that children might have genetic diseases	Yes	1851	90.96	χ^2^ = 1365.5, p < 0.001
	No	184	9.04	

In examining the factors influencing the recognition of the benefits of premarital screening tests, the logistic regression model demonstrated several significant associations. Individuals with secondary education levels showed a negative association (Estimate: − 0.7988, Std. Error: 0.2737, z-value: − 2.919, *p*-value: 0.00351), indicating a lower likelihood of recognising the benefits compared to those with higher educational levels. In contrast, having a positive family history was positively associated with the recognition of premarital screening benefits (Estimate: 0.43975, Std. Error: 0.15752, z-value: 2.792, *p*-value: 0.00524). Additionally, individuals with graduate-level education were more likely to acknowledge the importance of premarital screening tests (Estimate: 0.34919, Std. Error: 0.17327, z-value: 2.015, *p*-value: 0.04388). Interestingly, intermediate-level education also showed a significant negative association (Estimate: − 1.49306, Std. Error: 0.52898, z-value: − 2.823, *p*-value: 0.00476) (Supplementary Table 8).

Approximately 56.7% of the participants were aware of GC; however, 80.11% had never visited a GC clinic (Supplementary Table 21).

### Rate of CM and partner choice

**Fig. 2:**
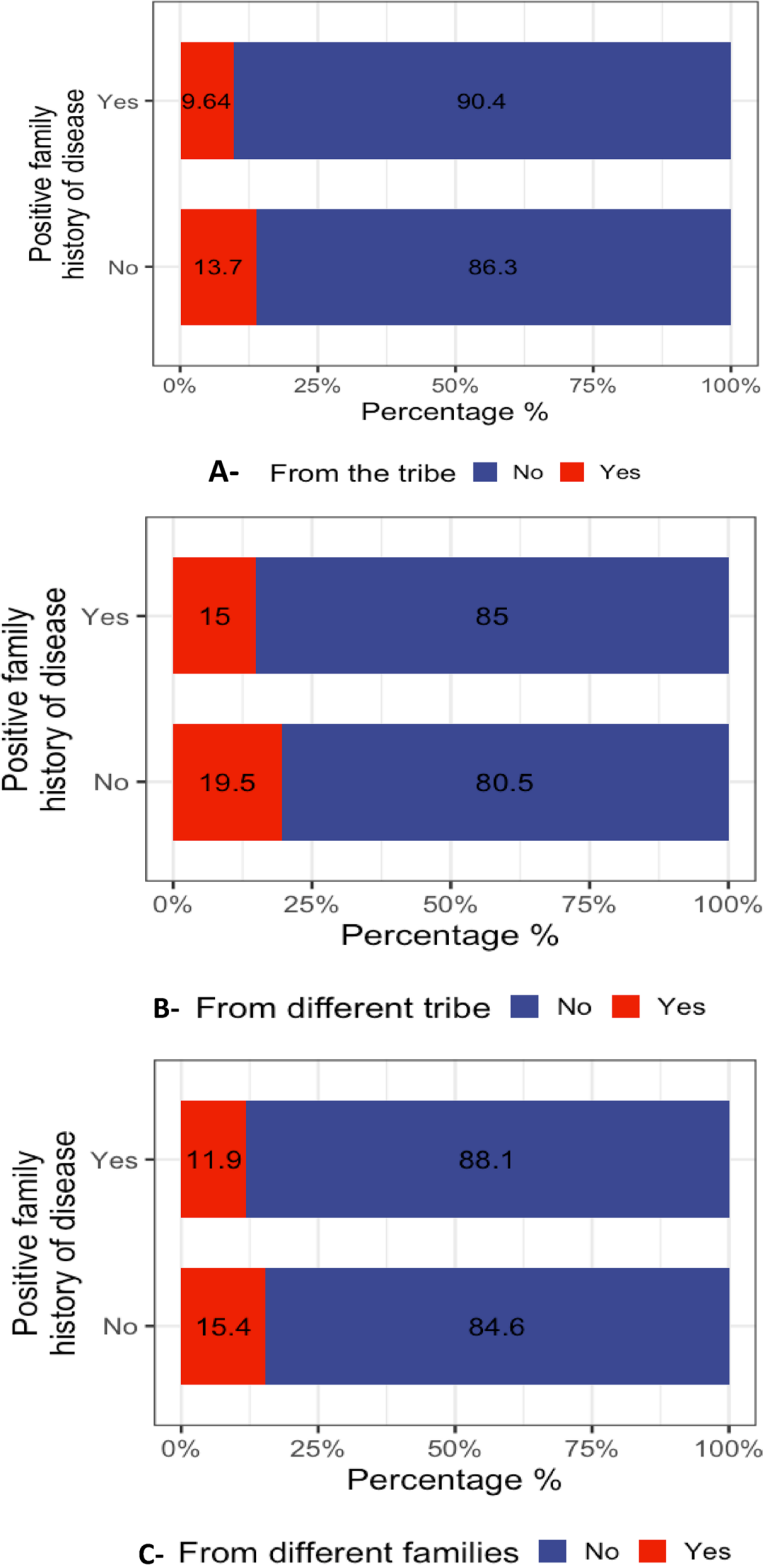
Partner kinship with/without a positive family history. **A** Participants with a history of genetic diseases were less likely to marry a partner from the same tribe compared to those without a history of genetic diseases (9.64% vs. 13.7%, respectively; *p*-value = 0.000139). **B** Participants with a positive family history declared that they married partners from different tribes compared to those with no family history of genetic diseases (15% vs. 19.5%, respectively; *p*-value = 0.000138). **C** Among participants with a positive family history, only 11% were married to partners from a different family compared to those with no family history of genetic diseases (15.4% with *p*-value = 0.000489)

About 22% of the participants were second or third-degree relatives, and 62.6% preferred arranged marriages or were already in an arranged marriage supplementary Fig. 1. A significant correlation was found between a positive family history and partnering choices. The correlation with the same tribe, different tribe or another family, and other variables regarding whether a partner is a relative (Table 2) was not significant, as depicted in Fig. 2 and Supplementary Table 27. The multivariable logistic regression analysis presents an association between partner relationships among individuals with a positive family history. A negative association was observed between the preference for arranged marriages and having a partner who was a third-degree relative, indicated by an estimate of − 0.295647 and a *p*-value of 0.126; it did not reach statistical significance. Notably, the strongest and statistically significant result was for individuals whose partners were from different families, showing a decisive negative preference towards arranged marriages, with an estimate of − 0.602510 and a highly significant *p*-value of less than 0.001. The analysis indicates a statistically nonsignificant weak trend for those who prefer to choose their partner over an arranged marriage, with an estimate of 0.050307 and a *p*-value of 0.711. Similarly, the preference for arranged marriages among individuals already married through arrangements, though higher, did not reach statistical significance, with an estimate of 0.199167 and a *p*-value of 0.285 (Supplementary Table 27).

### The desire to prevent inheritable diseases

Less than half of our participants risked having offspring with hereditary disorders (44.57%). The majority of respondents (96.12%) were willing to proceed with genetic testing if they or their partners had a history of genetic disease. An analysis of the influence of family history and individual history on the risk perception of genetic disorders was significant. The logistic regression model pinpointed the positive family history variable, denoting an affirmative response to possessing a family history of genetic conditions, as a significant factor (Estimate: − 0.56009, Std. Error: 0.23228, z-value: − 2.411, *p*-value: 0.0159). A positive family history of genetic disorders emerged as a significant predictor of increased awareness, although the effect was modest (Estimate: 0.34902, Std. Error: 0.15648, z-value: 2.230, *p*-value: 0.02572) (Supplementary Table 9).

**Fig. 3:**
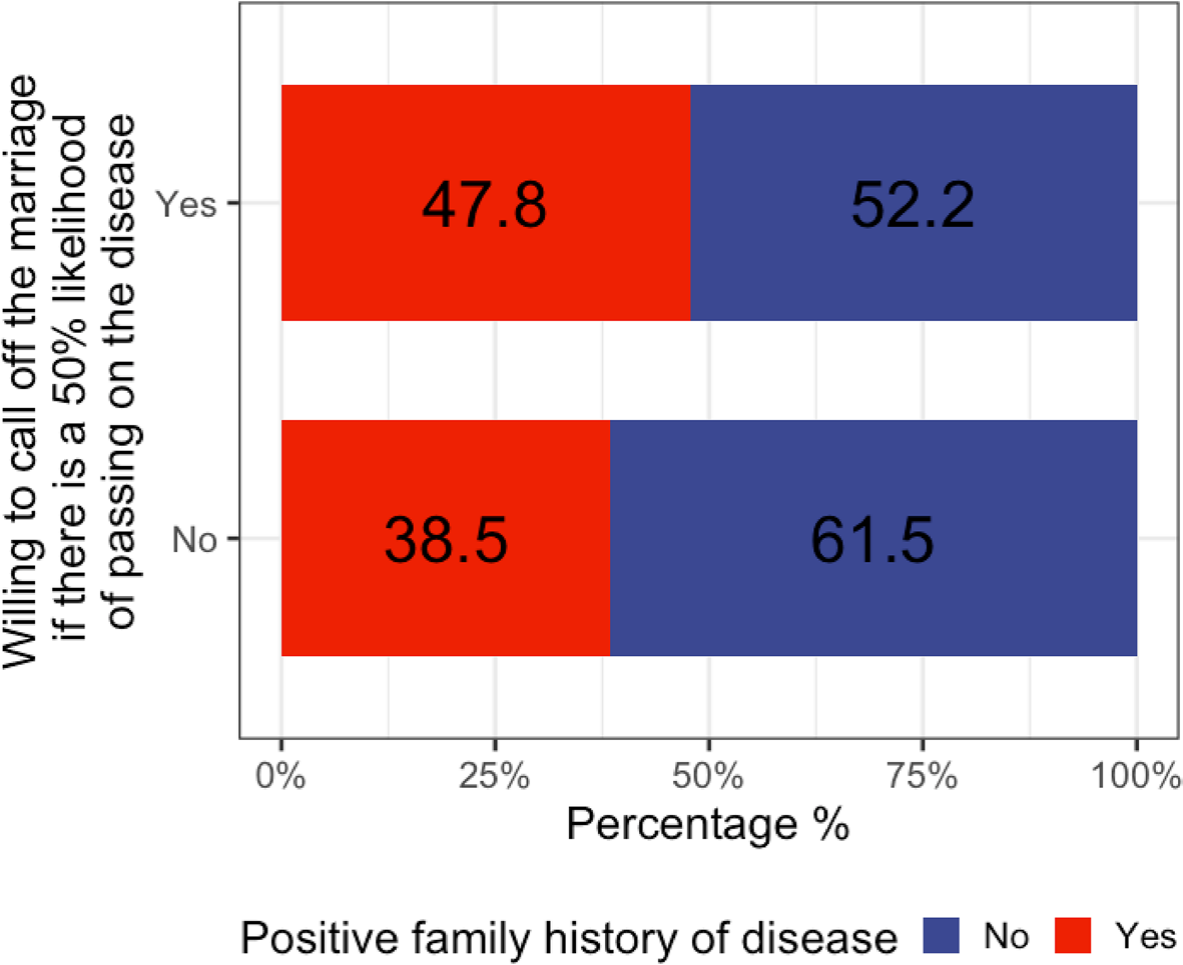
Participants decided to call off the marriage if they knew there was a 50% chance of passing on the genetic disease. This reveals that 47.8% of the participants with a positive family history were willing to call off a marriage, with a significance level of *p* = 0.0299

The vast majority of the participants (89%) would call off a wedding if they knew that each child would have a 50% chance or more of developing a genetic disease. Correlating the family history with this decision, we found that 47.8% had a family history of genetic diseases (Fig. 3). Between genetic disorders and societal perceptions, two logistic regression analyses found significant predictors. Individuals with an income of 10,000–15,000 SAR and those from the southern region were more likely to call off weddings due to genetic disease risk (Table 3). Having a graduate education or a positive family history slightly increased the likelihood of proceeding with the wedding. Conversely, graduate education decreased the fear of society’s perception, while residents of the western region showed increased concern. These findings highlight the influence of socio-economic and regional factors on attitudes towards genetic risks in marital decisions.

**Table 3:** Influence of Income, Education, Family History, and province on: ***“***Have you heard about genetic disease”(model 1), “Have you recognized the benefit of premarital screening tests” (model 2), “If you or your partner have or have a history of genetic diseases are you willing to test for it before marriage” (model 3), “I do not know about the role of a genetic counsellor” (model 4)

		**Model 1**		**Model 2**		**Model 3**		**Model 4**	
		OR	95% CI	OR	95% CI	OR	95% CI	OR	95% CI
Income	< 5,000 SAR^a^								
	5,000–9,999 SAR	0.70	(0.26, 1.14)	0.62	(0.3, 0.94)	0.26	(-0.08, 0.61)	0.15	(0.01, 0.28)
	10,000–14,999 SAR	1.11^b^	(0.59, 1.63)	0.77^b^	(0.43, 1.11)	0.06	(-0.27, 0.38)	0.1	(-0.03, 0.23)
	> 15,000 SAR	0.41	(-0.02, 0.84)	0.45	(0.13, 0.77)	0.37	(0.02, 0.71)	0.15	(0.02, 0.29)
Education	Bachelor^a^								
	Illiterate	11.78	(-870.96, 894.53)	11.35	(-524.06, 546.76)	13.28	(-2386.26, 2412.82)	12.28	(-312.46, 337.02)
	Elementary	-4.88^b^	(-6.11, -3.65)	-1.79	(-2.97, -0.62)	13.39	(-1149.13, 1175.92)	-1.5	(-2.66, -0.34)
	Intermediate	-2.52^b^	(-3.17, -1.87)	-0.36	(-1.41, 0.69)	13.4	(-566.49, 593.29)	-0.07	(-0.59, 0.44)
	Secondary	-0.17	(-0.61, 0.27)	-0.8^b^	(-1.07, -0.53)	-0.02	(-0.4, 0.36)	-0.06	(-0.21, 0.09)
	Graduate	0.54	(0.1, 0.98)	0.61	(0.28, 0.94)	-0.1	(-0.37, 0.18)	-0.45^b^	(-0.56, -0.35)
Family History	No^a^								
	Yes	0.50	(0.17, 0.83)	0.12	(-0.1, 0.35)	-0.56^b^	(-0.79, -0.33)	-0.09	(-0.18, 0)
Province	Central^a^								
	Eastern	-0.45	(-0.88, -0.02)	0.69	(0.27, 1.11)	0.98^b^	(0.5, 1.46)	0.13	(-0.01, 0.27)
	Northern	-0.46	(-0.96, 0.03)	-0.36	(-0.7, -0.01)	0.1	(-0.32, 0.53)	0.14	(-0.03, 0.32)
	Southern	1.08	(0.22, 1.94)	0.32	(-0.1, 0.75)	1.47^b^	(0.74, 2.21)	0.22	(0.06, 0.39)
	Western	-0.17	(-0.56, 0.21)	0.13	(-0.15, 0.41)	-0.18	(-0.45, 0.08)	0.37^b^	(0.25, 0.49)

OR: Odds Ratio, CI: Confidence Interval

^a^Reference Group

^b^Significant at a 5% leve

However, 92.9% of the participants understood the alternative options for preventing children from inheriting genetic diseases, and 46.93% were aware of the option of obtaining a PGD, while 94.3% considered visiting a GC and following the recommendations of the GC (Table 4). With regard to the medical termination of pregnancy (MToP) as a preventive measure, the logistic regression analysis scrutinising the determinants of MToP reveals significant gender- and education-related disparities. Males are less likely to opt for termination following a positive genetic test (Estimate =  − 0.373488, *p* < 0.0001), suggesting a strong gender-based divergence in responses to genetic risk. Educational attainment markedly influenced decisions, with graduates demonstrating a higher propensity for termination (Estimate = 0.472017, *p* = 2.65 × 10^−6^). Notably, the willingness to proceed with a marriage arrangement despite the potential of a genetic disorder was significantly less likely (Estimate =  − 0.616443, *p* = 0.000251), indicating a decisive stance against risking genetic disease propagation. Though integral to the model, factors such as consulting a GC, positive family history and preferences for marriage arrangement did not exhibit statistical significance in influencing termination decisions (Supplementary Table 29) and Supplementary Figure S.2.

**Table 4:** Participants’ Awareness of genetic testing

Category	Variable	N	%	Chi-square test
I don’t know if I have a genetic disease	Yes	1899	93.32	χ^2^ = 1527.4, p < 0.001
I don’t hear that my family has a genetic disease	Yes	915	44.96	χ^2^ = 21.97, p < 0.001
I don’t know about the genetic testing	Yes	1246	61.23	χ^2^ = 102.63, p < 0.001
I don’t know about the role of a genetic counselor	Yes	1114	54.74	χ^2^ = 18.304, p < 0.001
I don’t have enough money for genetic testing	Yes	534	26.24	χ^2^ = 459.5, p < 0.001

### Governmental regulations to avoid the spread of genetic diseases

Regarding reducing the social restrictions put in place within a tribe, 62.4% of participants agreed with the need to change restrictions, 91.3% agreed with the need for increased public awareness and improved knowledge of genetic disorders, and 87% agreed that more government regulations are needed to avoid passing genetic diseases from one generation to another (Table 5). However, no correlation was established between salary, education level, residential province and governmental role in reducing social restrictions and arranged marriages within tribal communities in the country.

**Table 5:** Participants’ Perception of genetic testing

I don’t feel that my marriage can affect my children in the future	Yes	1816	89.24	χ^2^ = 1253.3, p < 0.001
I thought premarital screening was enough to save my family from genetic diseases	Yes	1387	68.16	χ^2^ = 268.36, p < 0.001
Have you heard of PGD?	Yes	955	46.93	χ^2^ = 7.6781, p = 0.005
I am afraid of society’s perception if they know I have a genetic disease that can affect my children	Yes	1310	64.37	χ^2^ = 168.17, p < 0.001
Government regulation to avoid genetic diseases from being passed on in affected families	Encourage	1773	87.13	χ^2^ = 1121.9, p < 0.001
Saving my children	Encourage	1881	92.43	χ^2^ = 1465.6, p < 0.001
Understanding alternative options to avoid inherited diseases in children	Encourage	1892	92.97	χ^2^ = 1503.2, p < 0.001
Reduce social restrictions arranged within clan or tribe	Encourage	1272	62.51	χ^2^ = 127.31, p < 0.001
Health insurance covers genetic testing	Encourage	1504	73.91	χ^2^ = 465.22, p < 0.001
The price of the test	Encourage	648	31.84	χ^2^ = 268.36, p < 0.001
Awareness campaign to improve knowledge about genetic disease	Encourage	1859	91.35	χ^2^ = 1391.9, p < 0.001

Regarding genetic testing costs, 68.16% of participants were discouraged from testing because of cost, while 73.91% were encouraged to proceed when genetic testing was covered by the healthcare system. A regression analysis of factors affecting the perception of governmental regulations regarding the cost of genetic tests revealed several significant predictors. The level of education showed varying impacts on the perception of test pricing. Individuals with a secondary level of education were more likely to perceive genetic test pricing as favourable (Estimate: 0.322780, Std. Error: 0.156317, z-value: 2.065, *p*-value: 0.038932), while those with graduate-level education had an even stronger positive association (Estimate: 0.42925, Std. Error: 0.12941, z-value: 3.317, *p*-value: 0.00091) (Supplementary Table 26). Conversely, a positive family history was associated with a negative perception of pricing (Estimate: − 0.353448, Std. Error: 0.101274, z-value: − 3.490, *p*-value: 0.000483). Our analysis demonstrated that educational level plays a pivotal role in health insurance coverage for genetic testing. Individuals with a graduate education were significantly more likely to have insurance coverage for genetic testing (*p* = 0.00091). Those with only secondary education (*p* = 0.02901) and a positive family health history (*p* = 0.00629) were less likely to have such coverage. This suggests an educational disparity in accessing genetic testing through insurance.

### Perceptions of genetic disease and societal stigma towards hereditary disorders

Approximately 64% of participants feared society’s perceptions of genetic diseases that could affect their children. Our findings indicate that graduate education correlated with a reduced fear of societal perceptions of genetic disorders (*p* = 0.00871). Geographic variation was observed, with residents in the western region showing significantly greater concern about societal perceptions (*p* = 0.00934). This points to the influence of regional cultural and social factors in shaping attitudes towards genetic risks. All logistic regressions correlating variables with education level, income, presence of family history and residential province are detailed in Supplementary Tables 1–26. The output from each test is organised on individual sheets for clarity and ease of reference. Extra correlations were made with other variables in Supplementary Tables 27–29.

### ‘Perception Category

In the bivariate analysis, the "Perception Category" was a fixed variable to compare against the awareness variables and socioeconomic characteristics. Notable associations were observed in the perceptions related to marital impact on health, where the statement "I don’t feel that my marriage can affect my children’s health" was significantly associated with this category, yielding a Chi-square value of 328.37 (p < 0.001). Similarly, perceptions about the adequacy of premarital screening (“I thought premarital screening was enough to secure my children’s health”) also showed a strong linkage, with a Chi-square statistic of 331.84 (*p* < 0.001). The concern about societal judgment (“I am afraid of society’s perception if they knew about my children’s genetic issues”) marked the highest statistical significance, with a Chi-square of 561.35 (*p* < 0.001). In contrast, comparisons with demographic variables such as gender and age group revealed no significant associations (*p* > 0.75), emphasizing the prevailing influence of perception-related factors over these demographics within the study context.

Post-hoc logistic regression analyses were conducted using only variables that showed significant associations in the bivariate analysis. The results in Table 6 revealed that perceptions regarding marriage's impact on children's future health were strongly predictive. Specifically, respondents affirming that marriage can affect their children's health exhibited significantly higher odds of this perception in both unadjusted (OR = 2.56, 95% CI: 2.39–2.73) and adjusted models (OR = 2.60, 95% CI: 2.38–2.81). Similarly, beliefs about the sufficiency of premarital screening were significantly associated with increased odds in both unadjusted (OR = 1.92, 95% CI: 1.81–2.04) and adjusted analyses (OR = 2.11, 95% CI: 1.96–2.25). Additionally, concerns regarding societal perception of a genetic disease is known showed elevated odds (unadjusted OR = 2.66, 95% CI: 2.54–2.79; adjusted OR = 2.59, 95% CI: 2.44–2.73).

**Table 6:** Logistic Regression Modelling of Significantly Associated Awareness and Socioeconomic Factors with Perception Scores

		**Unadjusted Score (Model 1)**		**Adjusted Score** **(Model 2)**	
		OR	95% CI	OR	95% CI
Income	< 5,000 SAR^a^				
	5,000–9,999 SAR	-0.05	(-0.19, 0.1)	0.23	(0.03, 0.43)
	10,000–14,999 SAR	0.16	(0.02, 0.3)	0.46^b^	(0.27, 0.66)
	> 15,000 SAR	0.34^b^	(0.21, 0.48)	0.51^b^	(0.32, 0.70)
I don’t feel that my marriage can affect my children in the future	No^a^				
	Yes	2.56^b^	(2.39, 2.73)	2.6^b^	(2.38, 2.81)
I thought premarital screening was enough to save my family from genetic diseases	No^a^				
	Yes	1.92^b^	(1.81, 2.04)	2.11^b^	(1.96, 2.25)
I am afraid of society’s perception if they know I have a genetic disease that can affect my children	No^a^				
	Yes	2.66^b^	(2.54, 2.79)	2.59^b^	(2.44, 2.73)

*OR* Odds Ratio, *CI* Confidence Interval

^a^Reference Group

^b^Significant at a 5% level

Income levels also influenced perception scores significantly. For the income bracket of 10,000–14,999 SAR, the adjusted odds ratio was 0.46 (95% CI: 0.27–0.66), and for > 15,000 SAR, it increased to 0.51 (95% CI: 0.32–0.70), indicating stronger perceptions at higher income levels compared to the reference group.

## Discussion

The Saudi Premarital Screening Programme was established by the Ministry of Health of Saudi Arabia to identify and reduce the impact of various genetic and sexually transmitted infections in the Saudi population, particularly among high-risk groups, to support decision-making to limit the transmission of genetic diseases to their offspring. This programme was implemented because of the country’s high prevalence of CMs, which led to an increased incidence of genetic diseases being passed on.

In this study, we attempted to assess the awareness of genetic diseases and PMST, along with couples’ perceptions of genetic diseases before and after marriage and their attitudes towards PMST results and genetic counselling. Therefore, we sought to bridge the knowledge gap present in the existing literature on these topics.

A positive family history of genetic disorders emerged as a significant predictor of increased awareness, although the effect was modest. These findings underscore the critical role of educational attainment and familial experiences in shaping public knowledge of genetic disorders, which is pivotal for implementing effective genetic counselling and screening programmes in the community. When assessing the influence of family history and individual history on the risk perception of genetic disorders, individuals who acknowledged a family history of genetic diseases perceived reduced risk of these conditions for themselves, contrary to expected patterns. This counterintuitive result merits further investigation to understand the underlying causes, such as potential biases in risk self-assessments or a lack of understanding of genetic inheritance patterns. These insights are useful for developing educational strategies and genetic counselling services to address misconceptions and enhance the accuracy of individual risk perceptions of genetic epidemiology.

In examining the factors influencing the recognition of the benefits of premarital screening tests, individuals with higher educational levels were more likely to recognise the benefits compared to others. A positive family history was positively associated with the recognition of premarital screening benefits. These findings underscore the critical role of education and family health history in shaping attitudes towards premarital screening. This reinforces the need for targeted educational interventions.

In the current PMST, the diseases included are rather limited and need to be updated with the carrier screening programme to include the most common diseases in Saudi Arabia. Some countries with high CM rates of up to 25% and a high rate of genetic disorders, for example, Israel, have implemented an expanded PMST programme involving 22 genetic diseases, thereby reducing the incidence of genetic disorders, such as spinal muscular atrophy, by 57% [33, 34].

The Saudi population exhibited a favourable attitude towards PGD. Saudi families who had a child with thalassaemia were hesitant to have another child without a PGD [35]. The primary concern in Saudi Arabia was the access to pre-and post-genetic counselling services and PGD, which might not be funded by Saudi insurance or continue to be funded by a governmental body such as in the UK, France and the United States [36, 37]. There is a need for a robust system to regulate and determine the criteria for accepting a family for PGD, the genetic disorders that warrant PGD, and the number of cycles that an insurance company will fund, as done in other countries [36, 37].

In the investigation of factors related to governmental regulations affecting the pricing of genetic tests, the findings suggest that individuals with a family history of genetic conditions may perceive the tests as less affordably priced. These insights are critical for policymakers who must consider the impact of educational background and family history when regulating the genetic testing market to ensure equitable access. Another option to avoid having a child with a genetic disorder is prenatal diagnosis (PND). There is a demand to develop a national policy on heritable genetic disorders that warrant PND and the consequences among confirmed cases in terms of MToP. Although the MToP is regulated by religious authority or fatwa number 26307, which explains the conditions that warrant MToP, there is a need to unify the practice of MToP among Saudi health institutes. Hence, awareness campaigns must be implemented to change the Saudi population’s perspective towards marital trends. The burden of genetic diseases can be decreased by changing marriage patterns and introducing effective public education and screening programmes [38].

This study has several limitations. Data were exclusively collected via an online survey, potentially limiting the engagement of participants with lower education levels. A significant portion of the respondents hailed from the central region of Saudi Arabia. Despite efforts to engage participants from various regions, especially those with a higher prevalence of genetic diseases – via genetic clinics – participation rates from these areas remained low. Future efforts could integrate awareness campaigns about genetic diseases and the significance of family planning, alongside visits to genetic counselling centres, to enhance participation. The employment of random sampling, the Slovin formula for sample size determination and the adherence to specific time frame requirements also pose notable limitations to our survey’s methodology, impacting the generalisability and accuracy of our results. These measures, intended to increase the study’s feasibility, may introduce bias and constrain our capacity to reliably extend our findings to the larger population. Moreover, the use of the Slovin formula, while offering a practical solution for sample size calculation, may not fully capture the complexity and diversity of the target population. Our findings underscore the powerful influence of personal beliefs and economic status on health-related perceptions. This highlights the critical role these factors play in shaping health behaviour and awareness in the community, emphasizing the societal implications of our study.

The prevalence of CMs in Saudi Arabia is approximately 50%, contributing to a high rate of genetic disorders within the population. Despite increasing awareness among Saudis regarding inherited genetic disorders and the importance of PMSTs, there remains a need to address entrenched marital customs within communities. The pre-marriage screening programme should be reviewed and expanded to include molecular testing, particularly in families with a high prevalence of genetic disorders. Financial coverage should also be reviewed by health insurance and GC services. Insurance companies should also fund PGD and PND and develop a system to regulate them and unify the practices among Saudi health institutes.

## Data Availability

Data is available upon request. Please contact Dr.Mariam AlEissa at mmeissa@pha.gov.sa.
